# Editorial: New Hypervalent Iodine Reagents for Oxidative Coupling

**DOI:** 10.3389/fchem.2021.642889

**Published:** 2021-02-22

**Authors:** Toshifumi Dohi, Jian-Wei Han, Ravi Kumar

**Affiliations:** ^1^ College of Pharmaceutical Sciences, Ritsumeikan University, Shiga, Japan; ^2^ School of Chemistry and Molecular Engineering, East China University of Science and Technology, Shanghai, China; ^3^ Department of Chemistry, J. C. Bose University of Science and Technology, YMCA, Faridabad, India

**Keywords:** hypervalent compounds, iodine, reagent, oxidative coupling, synthetic application

In theory, oxidative coupling is a straightforward method for reducing the number of synthetic steps, avoiding the preparation of pre-activated substrates and less waste co-product generation by using metal salts. However, attempts at oxidative cross-coupling are challenging due to its limited synthetic applications and chemoselective issues. In recent years new oxidative coupling methods have emerged, using the C–H bond of the two substrates, and enabling the selective formation of cross-coupling products (Stuart and Fagnou, 2007).

One of the innovative research fronts in this area to have emerged in the past decade is the advance of oxidative coupling chemistry that uses hypervalent iodine reagents (Kita and Dohi, 2015; Yoshimura and Zhdankin, 2016), especially catalytic utilizations (Dohi and Kita, 2009; Dohi et al., 2013; Ito et al., 2013). This Research Topic discusses recent advancements of oxidative couplings and related reactions using hypervalent iodine compounds, highlighting the versatility of these reagents and their continuous development. Contributing to the recent advances in this area, the Research Topic includes contributions by experts exploring designs, preparations, reactions, mechanistic studies of hypervalent iodine compounds, and cooperative reaction systems with transition metals and photoredox catalysts, outlining how these synthetic applications can obtain useful organic molecules, such as pharmaceutical compounds.

In recent years there have been a number of successes in the participation of hypervalent iodine compounds in transition metal chemistry, which serve as strong electrophiles and powerful oxidizing agents, especially for the palladium-catalyzed couplings. The review by Shetgaonkar and Singh narrates recent advancements in this area, summarizing extensive work in the field of Pd-catalyzed C–H functionalizations, arylations, and other miscellaneous transformations with hypervalent iodine reagents and diaryliodonium(III) salts.

The synergistic combination of photoredox catalysis with hypervalent iodine reagents is one of many useful areas of organic synthesis. Chen et al. describe recent synthetic applications with visible-light-induced photoredox catalysis, focusing on the photochemical roles of hypervalent iodine reagents. However, a wide range of hypervalent iodine compounds still need to be explored under these conditions to bring out more synthetically useful transformations. We anticipate an expansion of this hot research area in the next few years.

In 2009, a novel cross-coupling method of heteroaromatic compounds was developed by exploiting the unique reactivities of diaryliodonium(III) salts (Kita et al., 2009). Since then, the chemistry of heteroaryliodonium(III) salts has undergone significant developments. They have proved to be useful reagents, acting as highly reactive electrophiles to bring about various transformations, such as NHC-catalyzed C-H bond arylation, Cu-catalyzed tandem arylation of indoles, and vicinal functionalization, etc. Another review by Takenaga et al. discusses the synthetic transformations mediated by heteroaryliodonium(III) salts with two classifications: 1) reactions utilizing the high reactivity of the hypervalent iodine(III) species; and 2) reactions based on unique and new reactivities not observed in other types of conventional diaryliodonium salts.

The Satkar et al. at Universidad de Guanajuato and Universidad Michoacana de San Nicolás de Hidalgo (Mexico) have contributed original research on diaryliodonium(III) salts, exploring their reaction with free-radical mechanisms for one-pot double arylation of naphthols. In this study, the new chemoselectivity pattern of the *C*- and *O*-centered naphthyl radicals toward the more electron-deficient hypervalent bond of the diaryliodonium(III) salts was observed for the first time. The naphthyl radicals were generated in the tetramethylpiperidinyl radical (TMP radical) reaction, resulting from the homolytic fragmentation of the precursor TMP_2_O. The generation of these radicals is supported by other spectroscopic, theoretical, experimental, and mechanistic studies.

Another promising study by Hashishin and Miyamoto et al. from Tokyo University presents research on the practical synthesis of useful alkynyliodonium(III) salt using calcium carbide as an ethynyl source. This study illustrates that the stannylation of calcium carbide followed by Sn–hypervalent iodine(III) exchange reaction cleanly affords the electrophilic ethynylating agent, ethynyl(phenyl)-λ^3^-iodane, in high yield. The use of inexpensive materials under easily operable reaction conditions and involving no special precautions, makes this protocol an effective, economical route to access unstable ethylnyl iodonium(III) salts.

As a part of the synthetic application of hypervalent iodine-mediated oxidative coupling in bioactive compounds, the Tan et al. research group summarizes efficient methodologies for the C3-H functionalization of quinoxalin-2(1*H*)-ones using hypervalent iodine reagents. Quinoxalin-2(1*H*)-one derivatives are known to show various biological activities and pharmaceutical properties. The review highlights the accomplishments of arylation, trifluoromethylation, alkylation, and alkoxylation of quinoxalin-2(1*H*)-ones with hypervalent iodine reagents by comparing reaction conditions and mechanisms.

Tan et al. report the diastereoselective α-acetoxylation of cyclic ketones by a binary hybrid system comprising a hypervalent iodine reagent and BF_3_•OEt_2_ Lewis acid. As they explain, this reaction involves an S_N_2 substitution mechanism *via* α-iodonium(III) ketone intermediate, and the diastereoselectivity mainly originates from thermodynamic control.

Iodine is an excellent promoter for several oxidative coupling reactions. Another contribution to this Research Topic by Li et al. demonstrates the I_2_-catalyzed oxidative coupling of *N*-tosylhydrazones with elemental sulfur to give 4-aryl-1,2,3-thiadiazoles. During the reaction, the DMSO oxidation of HI was used to generate the I_2_ exploiting dual properties of DMSO as oxidant as well solvent, an essential step in this reaction. Interestingly, this practical approach was extended to the synthesis of a neuroprotective compound. 

Finally, a study by Qiu et al. suggests 1-benzoyloxy-1,2-benziodoxol-3-(1*H*)-one (IBA-OBz) as a new efficient peptide coupling agent. The developed reaction system was successfully applied even to the solid-phase peptide synthesis and a pentapeptide leu-enkephalin in unprotected form. Density functional theory calculations have revealed that the rate-limiting step is nucleophilic attack of 4-dimethylaminopyridine (DMAP) onto IBA-OBz.

This Research Topic collection highlighs recent contributions on hypervalent iodine chemistry, providing informative research and presenting inspiring research in this area. Although there have been tremendous developments in hypervalent iodine chemistry in recent years, various areas still need to be investigated in detail.
